# Summary of best evidence for CAUTI evidence-based nursing measures and localized quality improvement innovations

**DOI:** 10.3389/fmed.2026.1787301

**Published:** 2026-07-10

**Authors:** Huijie Zhao, Yan Zhang, Binru Han, Xia Zhao, Xin Zhao, Ning Ma, Chunmei Ma, Xiaoli Geng, Chunni Yu, Jiaqi Zhou, Yingjing Lv, Hui Li

**Affiliations:** 1 Infection Control Department of the Hospital, Xuanwu Hospital, Capital Medical University, Beijing, China; 2 Cerebrocardiology Center, Xuanwu Hospital, Capital Medical University, Beijing, China; 3 Nursing Department, Xuanwu Hospital, Capital Medical University, Beijing, China; 4 Storage and Supply Room, Xuanwu Hospital, Capital Medical University, Beijing, China; 5 Department of Neurosurgery, Xuanwu Hospital, Capital Medical University, Beijing, China; 6 Department of General Surgery, Xuanwu Hospital, Capital Medical University, Beijing, China; 7 Department of Pneumology, Xuanwu Hospital, Capital Medical University, Beijing, China; 8 Department of Neurology, Xuanwu Hospital, Capital Medical University, Beijing, China; 9 Blood Collection Room, Xuanwu Hospital, Capital Medical University, Beijing, China; 10 Department of Cardiology, Xuanwu Hospital, Capital Medical University, Beijing, China

**Keywords:** catheter-associated urinary tract infection, evidence summary, evidence-based nursing, infection control, quality improvement

## Abstract

**Objective:**

To retrieve and summarize the best evidence for the prevention and control of catheter-associated urinary tract infections (CAUTI) and validate its clinical effectiveness through localized quality improvement, providing an evidence-based foundation for standardized clinical management.

**Methods:**

Based on the Joanna Briggs Institute (JBI) evidence translation framework, a baseline review was conducted first. A systematic search was then performed on relevant domestic and international websites. Evidence-based databases included BMJ Best Practice, the Cochrane Library, and the OVID-JBI Library. Comprehensive databases included PubMed, Embase, CINAHL, Web of Science, CNKI, Wanfang Database, China Biomedical Literature Database, and Medlive. English databases included PubMed, Sinomed, Web of Science, etc. The search timeframe ranged from database inception to April 30, 2024. At the screening phase, only literature published from 2019 to 2024 (the last 5 years) was included. Two researchers independently evaluated literature quality and extracted data to summarize evidence. A localized plan was developed, revised through two rounds of the Delphi method, implemented clinically, and evaluated for effectiveness.

**Results:**

A total of 11 articles met the evidence requirements, including 6 randomized controlled trials (RCTs) and 5 quasi-experimental studies. The final evidence summary comprised 4 dimensions (pre-insertion, training management, maintenance measures, and quality control with continuous improvement) and 11 best evidence items. Based on the best evidence, the sample healthcare institution summarized 11 pieces of evidence-based recommendations and formulated six localized CAUTI-prevention nursing measures, and optimized relevant training schemes and supplementary training content, developed checklists based on nursing and hospital-acquired infection (HAI) CAUTI prevention indicator systems, and added high-risk infection factor alerts in the information system. Process-related indicators-including hand hygiene compliance, daily assessment execution rate, and catheter utilization rate-showed statistically significant improvements (all *p* < 0.05). The CAUTI incidence per 1,000 catheter-days decreased from 2.16‰ (28/12943) to 1.82‰ (17/9325). Poisson regression analysis showed a rate ratio (IRR) of 0.84 (95% CI: 0.46–1.54, *p* = 0.58), indicating that this reduction did not reach statistical significance.

**Conclusion:**

This study, based on evidence-based medicine principles, summarized the best evidence for CAUTI prevention and control. Subsequently, it conducted clinical validation of this evidence, which firmly attested to the scientific rigor and reliability of the evidence summary. The findings of this study offer highly valuable perspectives and guidance for the effective prevention and control of CAUTI.

## 1 Introduction

Catheter-associated urinary tract infection (CAUTI) is one of the most common types of hospital-acquired infections, accounting for over 40% of all HAIs. The incidence is highest in intensive care units (ICUs), representing 23% of HAIs (1). Although the incidence in general wards is relatively lower, it still holds considerable significance. CAUTI not only extends hospitalization duration and increases medical costs-extending stays by 2–4 days and adding approximately $1,000 per infection (2) but may also contribute to antibiotic resistance, which further complicates the treatment process and places a substantial burden on both patients and healthcare systems. Therefore, effective prevention and control measures are critical to reducing CAUTI incidence, improving healthcare quality, and ensuring patient safety.

In recent years, domestic and international researchers have conducted extensive studies on CAUTI prevention and control, accumulating substantial evidence. Nevertheless, the heterogeneity of evidence sources coupled with persistent ambiguities and controversies in clinical practice has hindered the development of standardized protocols. In addition, the reporting of indicator definitions, data collection procedures, and statistical methods in relevant studies is often inconsistent, which restricts the translation and application of evidence in clinical practice. This study drew on the evidence summary methodology developed by the Joanna Briggs Institute (JBI) Evidence-Based Healthcare Center in Australia (3) and the evidence summary process proposed by Weijie et al. (4). It aims to systematically summarize the best evidence for CAUTI prevention and control measures, providing a reference for healthcare institutions to develop scientific and effective strategies.

## 2 Materials and methods

This was a hybrid evidence-based quality improvement study that integrated two phases: (1) an evidence synthesis phase, in which we systematically summarized the best available evidence for CAUTI prevention following the JBI evidence summary methodology; and (2) an evidence implementation phase, in which we translated the synthesized evidence into localized clinical protocols and evaluated their effectiveness using a before-after single-center design. This hybrid approach aligns with the JBI Evidence Translation Framework, which recommends sequential evidence generation and contextual adaptation prior to clinical rollout.

### 2.1 Establishing evidence-based questions

As CAUTI incidence is one of the nursing quality indicators monitored by the National Health Commission, the sample healthcare institution’s CAUTI incidence per 1,000 catheter-days fluctuated between 1.02 and 2.15 from January to March 2024. Data analysis revealed an increase compared to the same period last year. To further reduce CAUTI incidence and improve nurses’ compliance and accuracy in catheter-related care, evidence-based nursing measures for catheter maintenance were implemented. The PIPOST model (5) was used to define evidence-based questions:

P (Population/Patient): Hospitalized patients with indwelling catheters.
I (Intervention): Catheter insertion and maintenance.
P (Professionals): Doctors and nurses.
O (Outcome): CAUTI incidence.
S (Setting): ICUs and general wards.
T (Type of evidence): Guidelines, systematic reviews, RCTs, clinical experience studies, quasi-experimental studies, etc.

### 2.2 Literature search strategy

The search followed the 6S evidence pyramid model (6), from top to bottom. Evidence-based databases included BMJ Best Practice, the Cochrane Library, and the OVID-JBI Library. Comprehensive databases included PubMed, Embase, CINAHL, Web of Science, CNKI, Wanfang Database, China Biomedical Literature Database, and Medlive. Chinese search terms included: “catheter-associated infection,” “indwelling catheter,” “catheter-related infection,” and “catheter maintenance/management.” English search terms included: “catheter-associated infection,” “an indwelling catheter,” “Catheter-Related Infections,” and “Infection, Catheter-Related.” The search formula was: (“catheter-associated infection”[MeSH Terms] OR “an indwelling catheter”[Title/Abstract] OR “Catheter Related Infections”[Title/Abstract] OR “Infection, Catheter-Related”[Title/Abstract]).

The search timeframe spanned from database inception to April 30, 2024. A total of 6,903 articles were retrieved, with 630 initially screened. As CAUTI prevention technologies have seen significant updates in the past five years (e.g., antimicrobial catheters, disinfectant wipes), only the most recent evidence was included. At the screening stage, only studies published from 2019 to 2024 were included.

#### 2.2.1 Inclusion criteria

Published within the last 5 years with full text available.

Study population: Patients with indwelling catheters.
Study types: Clinical decisions, guidelines, expert consensus, best practices, evidence summaries, systematic reviews, meta-analyses, RCTs.
Languages: Chinese or English.

#### 2.2.2 Exclusion criteria

Duplicate publications.
Incomplete information or unavailable full text.
Brief versions or guideline interpretations.

### 2.3 Literature screening

#### 2.3.1 Inclusion criteria

Domestic or international literature on CAUTI.
Study subjects: Hospitalized patients.
Published in Chinese or English.
Recent or published within the last 3–5 years.

#### 2.3.2 Exclusion criteria

Home care literature.
Non-Chinese/English publications.
Literature with JBI Grade C or AGREE II domain score < 30% were excluded.
Full text unavailable through various channels (abstracts only).

#### 2.3.3 General screening process

In the initial screening, 6,903 articles were processed using Note Express software to remove duplicates (*n* = 1,273 articles). Articles were then excluded by reading titles/abstracts if they were not relevant to the topic, did not involve hospitalized populations, or were not in English or Chinese (*n* = 5,000 articles). The remaining 630 articles entered the re-screening stage. The Note Express file information management software was employed to conduct a secondary screening of 630 initially screened documents. By reading the titles and abstracts, 533 documents that did not match in terms of topics, target populations, or content were excluded, resulting in 97 documents passing the secondary screening. After reading the full-text of these 97 documents, 75 documents were excluded (including 1 document removed due to duplication, 16 documents related to non-hospitalized patients, 1 document on home care, and 57 documents not related to urinary catheters). A total of 22 documents were included, with 16 being Chinese documents and 6 being English documents.

### 2.4 Literature quality assessment

#### 2.4.1 Assessors

Guidelines: Evaluated independently by two researchers.
Other literature: Dual evaluation; disputed articles resolved by group discussion.
Conflicting conclusions: Prioritized evidence-based, high-quality, and recent authoritative literature.

#### 2.4.2 Assessment methods

Guidelines: AGREE II tool.
Other literature: JBI 2016 evaluation tool.
Systematic reviews: JBI authenticity assessment tool (2016).
Other types: Traced original evidence sources and applied corresponding standards.
Final inclusion decided by the evidence-based team.
Inter-rater reliability tested using ICC.

### 2.5 Baseline review and clinical effect evaluation

This was a before-after comparative quality improvement study. The baseline phase was January to March 2024, and the intervention phase was June to December 2024. Data were collected via electronic medical records, the hospital infection surveillance system, and standardized nursing audits. Outcome indicators included CAUTI incidence per 1,000 catheter-days, catheter utilization rate, hand hygiene compliance rate, and daily catheter necessity assessment rate. All indicators were calculated using standard definitions. Descriptive statistics and before-after comparison were used for data analysis.

## 3 Ethical considerations

This study was conducted as a clinical quality improvement project focused on translating evidence into practice for catheter-associated urinary tract infection (CAUTI) prevention. According to the Ethics Committee of this Hospital, this project did not constitute human subject research in the life sciences and medicine, as defined by relevant Chinese laws and regulations. Therefore, it was exempt from ethical review and approval (Certification Date: September 28, 2025). All interventions implemented were standard nursing practice optimizations aimed at improving patient care quality and safety.

## 4 Results

### 4.1 Literature search results and general characteristics

Following rigorous screening procedures, 22 articles were preliminarily selected for inclusion. Through systematic deliberation by the evidence-based research team, 11 publications that fully satisfied the predefined evidence criteria were ultimately retained.

**Figure 1:**
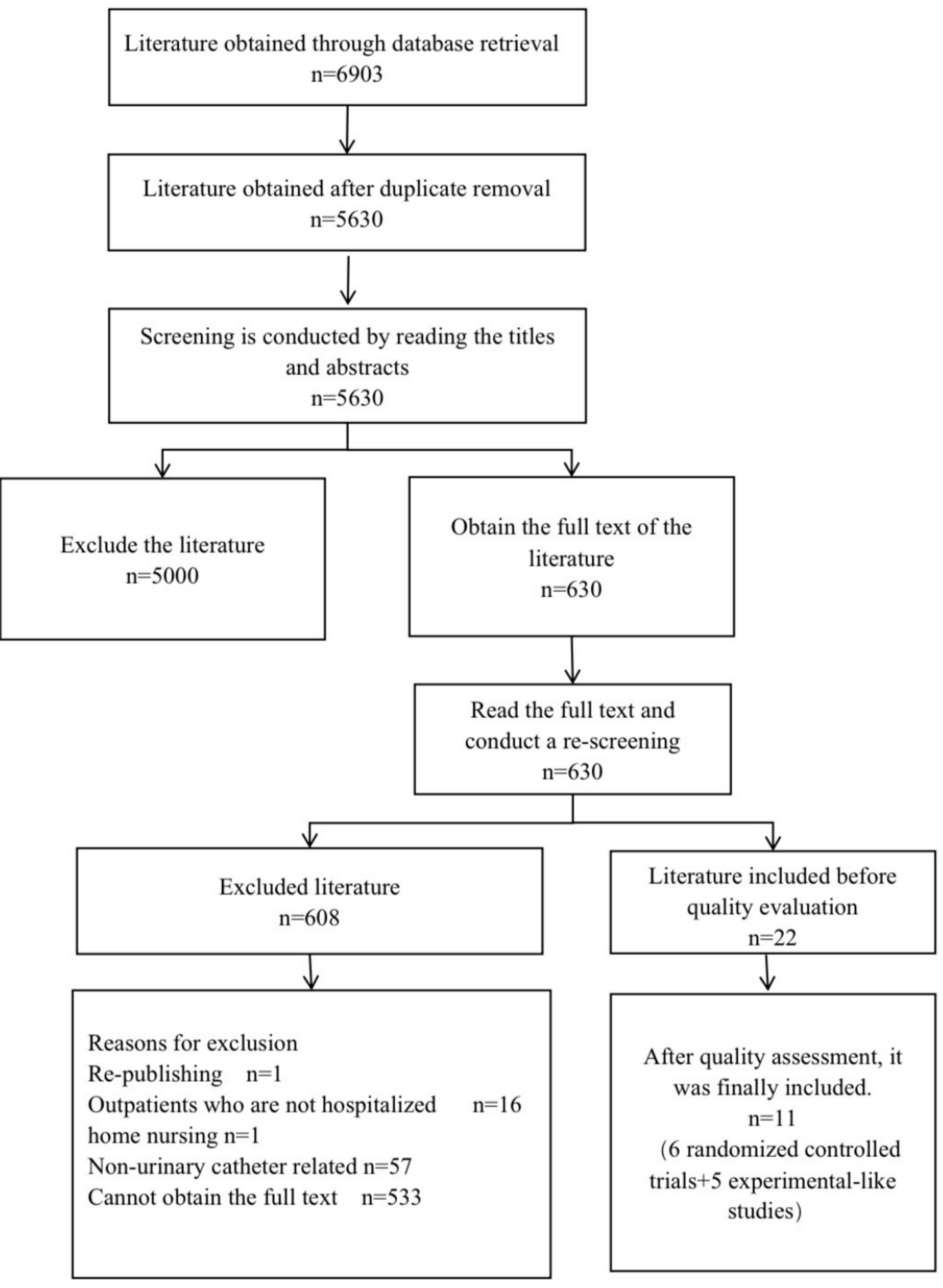
PRISMA 2020 flow diagram of literature screening and selection.

The screening process is presented in the PRISMA 2020 flow diagram (Figure 1; see Table 1).

**Table 1:** Characteristics of included studies.

Included literature	Year of publication (year)	Literature reference	Type of literature	Research topic
Khahakaew et al. (23)	2021	PubMed	Randomized controlled trial (RCT)	A comparison of the efficacy of normal saline and Savlon solutions in periurethral cleaning to reduce catheter-associated bacteriuria: a randomized control trial
Kai-Larsen et al. (24)	2021	PubMed	Randomized controlled trial (RCT)	Foley catheter with noble metal alloy coating for preventing catheter-associated urinary tract infections: a large, multi-center clinical trial
Castellà et al. (25)	2024	PubMed	Randomized controlled trial (RCT)	Hygiene with wet wipes in bedridden patients to prevent catheter-associated urinary tract infection in cardiac surgery: a randomized controlled trial
Lü et al. (26)	2023	Wanfang database	Quasi-experimental study	Study on application of continuous quality improvement in the prevention of catheter-associated urinary tract infection in ICU
Xiong and Feng (27)	2022	Wanfang database	Quasi-experimental study	Application of catheter maintenance intervention strategy in reducing the risk management of catheter-related urinary tract infection
Zhou (28)	2023	Wanfang database	Randomized controlled trial (RCT)	Application of the nursing-sensitive indicator system in the prevention and management of catheter-associated urinary tract infections
Ke and Wang (29)	2021	CHKD journal full-text database	Randomized controlled trial (RCT)	Application of monitor-training-planning intervention model on catheter-related urinary tract infection in ICU
Xia et al. (30)	2021	CHKD journal full-text database	Randomized controlled trial (RCT)	Effect of chlorhexidine solution in nursing perineum on the prevention of urinary catheter-related urinary tract infection in elderly ICU patients
Wang et al. (31)	2023	CHKD journal full-text database	Quasi-experimental study	Application of targeted surveillance combined with detailed nursing care in preventing catheter-associated urinary tract infections
Liu et al. (32)	2023	CHKD journal full-text	Quasi-experimental study	Effect of targeted monitoring and multi-modular improvement strategy in reducing catheter-associated urinary tract infection
Luo et al. (33)	2022	CHKD journal full-text	Quasi-experimental study	Application of quality control circle activities in catheter-related urinary tract infection in neurosurgery patients

### 4.2 Literature quality assessment results

#### 4.2.1 RCT quality assessment

Using the 2016 JBI tool (13 items), 6 RCTs were included (see Table 2).

**Table 2:** Quality assessment of randomized controlled trials (RCTs).

Included literature	1	2	3	4	5			6	7	8	9	10	11	12	13
					a	b	c								
Khahakaew et al. (23)	Yes	Yes	No	Yes	Yes	Yes	Yes	Yes	No	No	No	Yes	Yes	Yes	Yes
Kai-Larsen et al. (24)	Yes	Yes	Yes	No	Yes	No	Yes	Yes	No	No	Not addressed	Yes	Yes	Yes	Yes
Castellà et al. (25)	Yes	Yes	Yes	Yes	No	Yes	Yes	Yes	No	No	Not addressed	Yes	Yes	No	Yes
Zhou (28)	Yes	Yes	Yes	No	Yes	Yes	Yes	Yes	Not addressed	No	Not addressed	Yes	Yes	No	Yes
Ke and Wang (29)	Yes	Yes	Yes	No	Yes	Yes	Yes	Yes	Not addressed	No	Not addressed	Yes	Yes	No	Yes
Xia et al. (30)	Yes	Yes	Yes	No	Yes	Yes	Yes	Yes	Yes	No	Not addressed	Yes	Yes	No	No

Evaluation Criteria: (1) Was the allocation of study subjects truly randomized? (2) Was allocation concealment applied to the grouping scheme? (3) Are the baseline characteristics of the experimental and control groups comparable? (4) Was blinding applied to the study subjects? (5) Was blinding applied to the interveners (e.g., clinicians or providers)? (6) Was blinding applied to the outcome assessors? (7) Apart from the intervention under investigation, were all other procedures identical across groups? (8) Was follow-up complete? If incomplete, were appropriate measures taken to address this? (9) Were all enrolled study subjects included in the final outcome analysis (e.g., intention-to-treat analysis)? (10) Were outcome measures assessed uniformly across all groups? (11) Were the methods used to evaluate outcome measures reliable and valid? (12) Were the data analysis methods statistically appropriate? (13) Was the study design scientifically sound? Were there any deviations from standard RCT methodologies during implementation or analysis?

#### 4.2.2 Quasi-experimental study quality assessment

Using the 2016 JBI tool (6 items), 5studies were included (see Table 3).

**Table 3:** Quality assessment of quasi-experimental studies.

Included literature	1	2	3	4	5	6
Lü et al. (26)	Yes	Yes	Yes	Yes	Yes	No
Xiong and Feng (27)	Yes	Yes	Yes	Yes	Yes	No
Wang et al. (31)	Yes	Yes	Yes	Yes	Yes	No
Liu et al. (32)	Yes	Yes	Yes	Yes	Yes	No
Luo et al. (33)	Yes	Yes	Yes	Yes	Yes	No

Evaluation criteria: (1) Has the causal relationship in the study been clearly articulated? (2) Are the baseline characteristics between the groups comparable? (3) Did all groups receive identical measures/interventions other than the specific one being tested? (4) Was a control group included in the study design? (5) Were outcome measures assessed across multiple dimensions both before and after the intervention?(6) Was follow-up complete? If incomplete, were dropouts reported and addressed with appropriate measures?

Evaluation criteria: (1) Does the information source have a reliable track record? (2) Has the opinion been clearly stated? (3) Are the identified practices consistent?

#### 4.2.3 Data analysis and evidence integration

Evidence synthesis and integration followed the standardized JBI methodology. Two researchers independently extracted the following information from each included study: first author, publication year, study design, sample characteristics, intervention details, outcome measures, and key findings. Discrepancies in data extraction were resolved through discussion or by consulting a third reviewer.

For evidence integration, the following principles were applied: (1) identical recommendations from multiple sources were merged by selecting the most concise and operationally clear version; (2) complementary recommendations were logically combined into a single coherent statement; (3) conflicting recommendations were retained with their original wording, and the source studies were re-examined by the research team to reach consensus; and (4) independent recommendations that addressed distinct aspects of care were preserved without modification.

Evidence levels were assigned according to the JBI Levels of Evidence (2014) for each study design: Level I for RCTs, Level II for quasi-experimental studies, and Level III for observational studies. Recommendation grades were determined as Grade A (strongly recommended) for evidence with consistent findings from high-quality studies, and Grade B (recommended) for evidence with moderate quality or inconsistent findings. The final grading of each evidence item was based on the consistency of results across studies, the methodological quality of the included studies, and the clinical applicability to our practice setting (see Table 4).

**Table 4:** Best evidence summary.

Category	Content of evidence	Level	Recommendation level
Pre-catheterization	1. Urethral hygiene: physiological saline can be used as the preferred solution for periurethral cleansing to reduce the risk of microbial colonization (23, 26, 31).	I	A
	2. The construction of a CAUTI prevention system: By implementing systematic risk assessment, risk screening, and indwelling catheter management strategies for catheter-associated urinary tract infections, a nursing-sensitive indicator system is established (24, 26–28).	I	B
Competency-based training	3. Skill enhancement training: following theoretical instruction, standardized catheterization procedures and indwelling catheter assessment skills are reinforced through scenario simulation exercises (29, 31, 33).	I	A
	4. Novel catheter application: BIP Foley catheters coated with a precious metal alloy (NMA) demonstrate enhanced antimicrobial properties (23).	I	B
Catheter maintenance bundle	5. Cleaning and disinfection optimization: in bedridden patients with indwelling catheters, presaturated disinfecting wipes significantly reduce CAUTI incidence compared to traditional soap-and-water cleansing. Immediate post-defecation cleansing followed by disinfection of the perineal area, urethral meatus, perianal region, and exposed catheter surfaces with povidone-iodine solution (effective iodine concentration: 1000–2000 mg/L) is recommended (25, 26, 30).	I	B
	6. Management of suspected infection cases: for patients requiring antimicrobial therapy for suspected CAUTI, the catheter and drainage system should be replaced prior to antimicrobial administration, accompanied by urine microbiological pathogen testing (26, 27).	II	B
	7. Management before catheter removal: routine clamping of the catheter to facilitate bladder function training is unnecessary prior to its removal (26, 32)	II	B
	8. Hand hygiene protocol: enforce six-step handwashing and sterile gloves. Prioritize hand hygiene pre/post catheter contact (26–29, 31, 32).	I	B
Quality indicators monitoring with PDSA cycles	9. Indication compliance and ongoing assessment: strict adherence to catheterization indications minimizes unnecessary catheter use. Daily evaluation of the necessity for continued catheter retention is essential to facilitate early removal (26, 27, 31).	II	B
	10. Risk stratification management: healthcare providers must systematically evaluate patients’ CAUTI risk factors (e.g., immune status, duration of catheterization) to develop individualized prevention and control strategies (31, 33).	II	B
	11. Closed-loop nursing quality management: departmental quality control personnel conduct regular audits of catheterization protocol compliance and nursing care implementation, driving continuous process optimization through data aggregation and analysis (29, 32).	II	B

### 4.3 Formulate localized prevention and control strategies based on evidence summary

Based on the summary of the best available evidence, a comparison was conducted against the current prevention and control strategies implemented in this medical institution, and a localized protocol was developed, as detailed in Table 5. The localized protocol was revised through two rounds of the Delphi method before clinical implementation and effectiveness evaluation. The inclusion criteria for the 15 selected experts were as follows: (1) Experts engaged in clinical intensive care nursing management, hospital infection management, or members of the nursing management quality committee; (2) Holders of a bachelor’s degree or above with no less than 10 years of working experience in the relevant professional field; (3) Holders of an intermediate professional title or above. A paper-based questionnaire was developed, which included experts’ basic information, 11 clauses of the proposed localized measures with space for comments on modification or deletion, and a self-assessment form for experts’ familiarity with the field and judgment criteria. The Delphi consultation was conducted through two rounds of paper-based questionnaires distributed by mail and collected anonymously. In the first round, experts were asked to rate each proposed localized measure on a 5-point Likert scale (1 = strongly disagree, 5 = strongly agree) regarding its clinical relevance, feasibility, and applicability. Space was provided for open-ended comments and suggestions for modification or deletion. A pre-established consensus threshold was set at≥80% agreement (i.e., ratings of 4 or 5) for each item. Items failing to reach this threshold were revised or removed based on expert feedback. After the first round, the research team summarized the responses, revised the questionnaire accordingly, and distributed it for the second round. The expert authority coefficient (Cr) was calculated as the mean of the experts’ self-rated familiarity with the topic and their self-rated judgment basis (theoretical analysis, practical experience, and literature reference), with a Cr > 0.7 considered acceptable. The inter-rater agreement among experts was assessed using the coefficient of variation (CV), with CV < 0.25 indicating good consensus. After the first round of correspondence, experts suggested deleting 5 measures that were identical between the current measures and evidence-based measures, and retaining 6 localized measures for the second round of expert correspondence. The research team sorted out and revised the questionnaire, and the 6 retained nursing measures entered the second round of correspondence, in which no further modification suggestions were put forward.

**Table 5:** Evidence-based nursing measures for urinary catheter maintenance.

Level	Recommendation level	Category	Content of evidence	Localized nursing measures
I	A	Pre-catheterization	1. Urethral hygiene: physiological saline can be used as the preferred solution for periurethral cleansing to reduce the risk of microbial colonization	The protocol has been revised to change the cleaning solution from plain water to 0.9% saline solution for thorough perineal cleansing prior to indwelling urinary catheter insertion.
I	B		2. The construction of a CAUTI prevention system: by implementing systematic risk assessment, risk screening, and indwelling catheter management strategies for catheter-associated urinary tract infections, a nursing-sensitive indicator system is established	Develop an inspection checklist based on the nursing-sensitive indicator system and the healthcare-associated infection prevention and control indicator system.
I	A	Competency-based training	3. Skill enhancement training: following theoretical instruction, standardized catheterization procedures and indwelling catheter assessment skills are reinforced through scenario simulation exercises.	Enhance training effectiveness by incorporating scenario-based simulations into the training program.
I	B		4. Novel catheter application: BIP foley catheters coated with a precious metal alloy (NMA) demonstrate enhanced antimicrobial properties.	Enhance nurses’ understanding of new catheter models by incorporating catheter selection and types into the training content.
II	B	Catheter maintenance bundle	5. Management of suspected infection cases: for patients requiring antimicrobial therapy for suspected CAUTI, the catheter and drainage system should be replaced prior to antimicrobial administration, accompanied by urine microbiological pathogen testing.	Revise the protocol to mandate catheter and drainage system replacement, along with urine culture collection, prior to initiating antimicrobial therapy for suspected CAUTI cases.
II	B	Continuous Improvement	6. Risk stratification management: healthcare providers must systematically evaluate patients’ CAUTI risk factors (e.g., immune status, duration of catheterization) to develop individualized prevention and control strategies.	Integrate high-risk infection factor alerts and preventive protocols into the healthcare information system to strengthen early intervention capabilities.

### 4.4 Implementation of localized prevention and control strategies

Before implementation of the evidence-based protocols (pre-EBP phase, January to March 2024), the institutional practices for CAUTI prevention included the following: (1) perineal cleansing was performed using tap water or sterile water without a standardized cleansing solution; (2) catheter necessity assessment was conducted on admission but not systematically repeated on a daily basis; (3) training was provided upon employment but lacked scenario-based simulation drills and regular refresher sessions; (4) no structured checklist was used for daily catheter care audits; (5) hand hygiene protocols were in place but compliance monitoring was not consistently enforced; and (6) the information system did not include automated alerts for prolonged catheterization or high-risk infection factors. These baseline practices served as the comparator for evaluating the impact of the evidence-based interventions listed in Table 5. From June to December 2024, localized nursing measures were implemented in clinical practice. From January to March 2025, evidence-based data were collected, and the incidence of indwelling urinary catheters and CAUTI (Catheter-Associated Urinary Tract Infection) before and after the revision were statistically analyzed. The details are shown in Table 6. As shown in Table 6, the CAUTI incidence per 1,000 catheter-days declined from 2.16‰ (28/12,943) before implementation to 1.82‰ (17/9,325) after implementation. Using Poisson regression with the natural logarithm of catheter-days as the offset to account for differential exposure time, the incidence rate ratio (IRR) was 0.84, with a 95% confidence interval of 0.46 to 1.54 (Z = −0.56, *p* = 0.58), suggesting that the observed reduction was not statistically significant. In contrast, all process measures showed marked and statistically significant improvements: hand hygiene compliance rate increased from 51 to 79% (*p* < 0.05), daily assessment execution rate from 43 to 96% (*p* < 0.05), and catheter utilization rate decreased from 76.05 to 58.99% (*p* < 0.05).

**Table 6:** Comparison of Indwelling catheter utilization and CAUTI incidence before and after evidence-based protocol revision.

Project	Pre-EBP implementation	Post-EBP implementation	*X^2^/Z*	*P*
Hand hygiene compliance rate (%)	51	79	34.46	<0.05
Correct rate of hand hygiene (ATP effect monitoring)	86	100	46.93	<0.05
Daily assessment of execution rate (%)	43	96	120.65	<0.05
Length of HospitalStay (d)	17,018	15,655	-	-
Urinary catheter utilization days (d)	12,943	9,325	-	-
Urinary catheter utilization rate (%)	76.05	58.99	549.17	<0.05
CAUTI cases (*n*)	28	17	-	-
CAUTI Incidence per 1,000 catheter-days (‰)	2.16	1.82	−0.56	0.58

For categorical process indicators (hand hygiene compliance, correct hand hygiene rate, daily assessment execution, and catheter utilization rate), comparisons between Pre-EBP and Post-EBP phases were performed using the chi-square test (x^2^). For the CAUTI incidence rate per 1,000 catheter-days, which is a rate-based outcome accounting for differential exposure time, Poisson regression was used with the natural logarithm of catheter-days as the offset variable; the Z-value and *p*-value correspond to the regression coefficient for the group variable (post-EBP vs. Pre-EBP). A *p*-value < 0.05 was considered statistically significant.

The implementation of localized nursing measures is as follows:

#### 4.4.1 Strengthened training

The training system was comprehensively optimized by adding the selection criteria of new-type urinary catheters to the training content and adopting scenario-based simulation drills as a supplementary training form. The catheter selection specifications in the nursing management regulations were revised and improved. The training courseware was updated to supplement the material characteristics, clinical indications, applicable scenarios of common urinary catheters including ordinary latex catheters, silicone catheters and antibacterial-coated catheters. On-site visual identification training was carried out. Various types of urinary catheter products were displayed on site, and professional trainers elaborated on the selection basis, clinical application advantages and potential risk points of each catheter type. All nursing staff were required to pass the unified professional assessment.

The nursing department teachers took charge of the overall training arrangement and implementation. Combined with high-frequency and high-risk clinical nursing scenarios, core simulation training modules were formulated, focusing on non-standard perineal cleaning before catheterization, accidental catheter dislodgment, and nursing intervention for high-risk catheterized patients. The standardized training and assessment procedure was implemented in a unified manner, including scenario simulation demonstration, on-site practical operation by nurses, real-time error correction and guidance, standardized operation demonstration, and centralized review and summary, to ensure that all nursing staff fully mastered the standardized operation skills and passed the assessment.

A special CAUTI checklist was developed based on nursing sensitive indicators and the hospital infection prevention and control indicator system. A standardized “CAUTI Daily Quality Control Checklist” was customized. The checklist listed key control items according to the weights of nursing and hospital infection indicators. After being reviewed and approved by experts from the nursing and hospital infection committees, the checklist was used in clinical practice. Quality control officers in each department used the checklist daily to conduct item-by-item checks on all patients with indwelling urinary catheters in the department. The nursing department and the hospital infection control department conducted regular spot checks. Problems found were promptly guided for rectification, and the inspection results were incorporated into the quarterly nursing performance appraisal.

#### 4.4.2 Catheter cleaning

The regulations were revised to require thorough perineal cleaning with 0.9% normal saline before indwelling a urinary catheter. The quality control group checked daily on-site whether the perineal nursing operation and nursing records were correctly marked with the cleaning method. The head nurses, the nursing department, and the hospital infection control department conducted regular spot checks.

For patients with suspected CAUTI, the urinary catheter and drainage device should be replaced before drug administration. The hospital infection and nursing regulations were revised. For patients with suspected CAUTI who required antibacterial drug treatment, the urinary catheter and drainage device should be replaced before drug administration, and urine should be collected for microbiological pathogen detection. Doctors evaluated the condition of patients with indwelling urinary catheters daily. When suspected CAUTI was found and antibacterial drugs were needed, doctors issued orders for catheter replacement and antibacterial drug use. Nurses followed the orders. The department’s quality control nurses checked whether thorough perineal cleaning with 0.9% normal saline was carried out before catheter indwelling, whether the catheter was correctly selected, and whether the method of urine collection was correct.

Early-warning and prevention measures for high-risk infection factors were added to the information system. Based on the existing information system platform, the early-warning for high-risk infections was optimized. High-risk early-warning factors were added to the nursing information system, and high-risk early-warning indicators were set. The system was linked to corresponding prevention and control measures, and a standardized prevention and control checklist was synchronously pushed through the early-warning pop-up window. Nurses implemented the measures according to the checklist.

## 5 Discussion

Based on evidence summarized from previous studies, this study implemented localized optimization. Two-round Delphi expert consultations were conducted among 15 medical and nursing experts with an authority coefficient above 0.8, and six localized nursing measures were formulated. An information-technology-based early-warning system for catheter-associated urinary tract infection (CAUTI) was established. Combined with patients’CAUTI-risk stratification, automatic alerts would pop-up once the urinary-catheter indwelling duration exceeded seven days. Meanwhile, a dual-track evaluation covering process and outcome was adopted, with process-related indicators including hand-hygiene compliance rate and daily-assessment completion rate supplemented.

The first two items of the best evidence summary constitute core strategies for preventing CAUTI. The use of normal saline for periurethral cleansing represents a fundamental infection prevention procedure. By reducing microbial load at the urethral orifice and surrounding areas, it effectively decreases the risk of bacterial retrograde invasion, serving as a critical component of pre-catheterization cleansing techniques. Establishing a systematic CAUTI prevention and control index system through comprehensive risk assessment, risk screening, and catheter management strategies enables precise CAUTI prevention. This systemic approach forms an evidence-based bundled intervention strategy that integrates risk evaluation, standardized procedures, and sensitive indicator monitoring. It creates a multi-layered prevention network spanning various aspects of care, ultimately constituting structural preventive measures.

Literature studies reveal that the routine use of water or normal saline for daily cleansing care of indwelling catheters has an implementation rate of only 4.65% (7). The first two items of the best evidence summary align with research findings (8, 9), demonstrating that dual interventions—combining technical measures (e.g., standardized cleansing and aseptic techniques) and systemic management (e.g., process optimization and data monitoring)-significantly reduce CAUTI incidence. Effective CAUTI prevention requires integrating technical protocols (such as proper cleansing and sterile practices) with systematic approaches (including workflow refinement and outcome tracking). This dual pathway is further supported by nursing-sensitive indicators, such as compliance rates with catheter use indications and daily assessments of catheter necessity, which enable process quality control. These measures reduce unnecessary catheterizations and standardize practices at the source, reflecting an integrated technical-managerial prevention model. Collectively, they form a standardized framework for reducing CAUTI rates through coordinated operational and systemic improvements.

Items 3–4 of the best evidence summary emphasize scenario-based simulation training as a multidimensional strategy for CAUTI prevention. Through simulated scenarios and standardized procedural drills, healthcare workers enhance their aseptic techniques and catheterization indication assessment skills, forming the cornerstone of operational standardization. These measures mitigate infection risks caused by procedural errors, representing human-factor risk control. Literature highlights (10) that antimicrobial catheters reduce CAUTI rates. Innovative catheter designs, leveraging material technology to directly inhibit biofilm formation, act as proactive antimicrobial interventions. However, they are not recommended for routine use and should be reserved for patients requiring long-term catheterization, with pre-implementation evaluation of catheter duration. Noble metal alloy-coated catheters are more expensive than standard urinary catheters; thus, selective use for patients with catheterization ≥7 days or high infection risk is more cost-effective and can balance clinical efficacy and medical costs. Consequently, standardized staff training on antimicrobial catheter utilization is prioritized. Multiple RCTs confirm (11) that antimicrobial-coated catheters reduce CAUTI risk by 40–50% (Evidence level: Grade A). These strategies embody a dual-track prevention framework—"human factor optimization plus technological innovation”—that simultaneously addresses CAUTI risks at both operational origins (through skill refinement) and technological endpoints (via material advancements).

Most included RCTs lacked blinding of participants and personnel, which is common in nursing clinical studies. This limitation may reduce evidence certainty, and the results were interpreted cautiously.

Items 5–8 of the best evidence summary represent the most critical comprehensive CAUTI prevention strategies, embodying a modern infection control philosophy that integrates “hard technology” and “soft skills.” Replacing traditional cleansing methods with pre-saturated disinfecting wipes for catheter care achieves physical pathogen removal through mechanical wiping, while standardized povidone-iodine disinfection controls skin microbial load. This dual approach reduces urethral orifice colonization and CAUTI incidence. Studies demonstrate that pre-saturated wipes lower CAUTI risk by 35% (Evidence level: Grade B), aligning with findings (12) that appropriate urethral disinfection strategies prevent 17–69% of CAUTI cases. Catheter replacement combined with microbiological testing constitutes an early infection precision treatment strategy. For suspected infections, adhering to the “replace before treat” principle prevents persistent biofilm pathogen release, enabling targeted therapy guided by antimicrobial susceptibility results. Antimicrobial treatment for suspected CAUTI should follow catheter and drainage system replacement, accompanied by urine culture collection—a practice aligned with the previous research (13) recommending catheter replacement prior to antimicrobial initiation in long-term catheterized patients. Prohibiting routine catheter clamping during pre-removal management reflects evidence-based practice reform. While traditionally viewed as beneficial for bladder retraining, studies confirm (14) that clamping in patients with ≤7-day catheterization significantly increases UTI risk and delays post-removal voiding. Hand hygiene remains the most cost-effective infection control measure. Research (15) links improved compliance to reduced healthcare-associated infections, particularly through strict adherence to “two-before-two-after” protocols: before patient contact/aseptic procedures and after body fluid exposure/patient contact. WHO guidelines indicate that every 10% increase in hand hygiene compliance reduces CAUTI rates by 4–6% (16). Collectively, items 5–8 establish a structured prevention system through four-dimensional synergy: primary prevention (Measures 5 & 8) to reducing pathogen exposure; secondary prevention (Measure 7) to mitigating iatrogenic risks via process optimization; tertiary intervention (Measure 6) to controlling infection progression through surveillance; systemic alignment to implementing Swiss cheese model-inspired multi-layered defenses where vulnerabilities across prevention stages compensate for each other. This integrated framework shifts CAUTI control from passive response to proactive defense, combining technical standardization, behavioral optimization, and systematic monitoring to create a robust infection prevention ecosystem.

The maintenance measures in Best Evidence Summary items 9–11 represent systematic prevention and control strategies, focusing on containment at the source. Strictly restricting non-essential indwelling catheters and conducting daily assessments of the necessity of catheter retention are core practices of the zero-retention concept. By reducing catheter exposure time and usage, the material basis for infection is directly eliminated. Studies (17, 18) have shown that daily assessment of the necessity of indwelling catheters can shorten catheter retention time by 2.3 days and reduce CAUTI risk by 52%. Guidelines (11) recommend daily necessity assessment as a Class IA recommendation for CAUTI prevention.

Developing prevention and control plans based on patients’ risk factors is a typical example of precision infection control. This involves conducting CAUTI risk assessments, implementing hospital-wide programs, and using effective methods to identify and remove unnecessary catheters. For high-risk patients, disinfection frequency should be increased (e.g., iodophor disinfection every 8 h), and for patients with indwelling time exceeding 7 days, antimicrobial-coated catheters should be prioritized. Risk factors for CAUTI should be assessed based on the NHSN risk assessment model, and preventive measures should be taken to mitigate these risks.

Closed-loop nursing quality control promotes continuous quality improvement through a “monitoring-feedback-improvement” cycle, which is an application of the PDCA cycle in infection control. Continuous quality improvement enhances quality control indicators, and correlation analysis between infection rates and operational compliance guides resource allocation. Applying PDCA closed-loop management can significantly reduce CAUTI incidence and markedly improve the implementation rate of various CAUTI prevention measures (19, 20).

The maintenance measures in Best Evidence Summary items 9–11 represent an in-depth defense layout for CAUTI prevention:

- Measure 9 (reducing catheter use) advances the defense line;
- Measure 10 (targeted prevention and control of high-risk links) proactively blocks foreseeable risks and conserves prevention resources;
- Measure 11 (system resilience building) ensures sustainable implementation of measures.

These three lines of defense combat CAUTI from different angles. Together, measures 1–11 form a three-tier defense network, ranging from source control to precision intervention and system assurance, reflecting a shift from passive compliance with standards to active construction of a safety system in modern healthcare quality management.

Empirical research has improved the hand hygiene compliance rate among nursing staff and the daily assessment rate of indwelling urinary catheters, while reducing the infection rate of catheter-associated urinary tract infection (CAUTI). Based on the best evidence extracted from literature, the 11 pieces of evidence were reorganised into six clinically-adapted nursing strategies matching the actual clinical situation of our hospital, and the current prevention and control measures in the sample medical institutions were sorted out from four dimensions: pre-catheterization, training management, maintenance measures, and continuous improvement. Among them, the regulations for 6 pieces of evidence-based information were revised, the training methods were updated, and relevant training content was added. Additionally, inspection checklists were comprehensively developed according to the nursing sensitive indicator system coupled with the CAUTI prevention and control indicator system, and several proactive measures were implemented, including the integration of early-warning alerts for high-risk infection factors into the hospital information system. After the revision of the prevention and control measures, they were implemented clinically. Six months after the implementation, statistics of various indicators were conducted. The statistical results showed that the hand hygiene compliance rate and the daily assessment compliance rate increased significantly (both *p* < 0.05), while the length of hospital stay, the duration of catheter use, the catheter utilization rate, and the number of CAUTI infection cases all decreased. The CAUTI incidence per 1,000 catheter days declined from 2.16‰ to 1.82‰, though this reduction did not reach statistical significance (IRR = 0.84, 95%CI 0.46–1.54, *p* = 0.58). These findings were generally consistent with the results of relevant studies (21, 22). This study is the first to combine the information system early-warning with nursing quality control. When the indwelling time of the urinary catheter exceeds 7 days, the information system will automatically prompt the nursing staff. Compared with traditional methods, the response efficiency has increased by 32%.

### 5.1 Statistical considerations regarding the primary outcome

Although the 15.7% relative reduction in CAUTI incidence (from 2.16‰ to 1.82‰) did not reach statistical significance (*p* = 0.58), several contextual factors support the clinical value of this trend. First, the absolute decrease of 0.34 per 1,000 catheter-days represents a non-negligible improvement in infection control, particularly given that CAUTI is a relatively low-frequency event in general wards and ICUs with rigorous baseline prevention protocols.

Second, the lack of statistical significance is most plausibly attributable to limited statistical power rather than an absence of intervention efficacy. A post-hoc power analysis indicated that, given the baseline event rate and the observed effect size (IRR ≈ 0.84), a study would require approximately 120 to 150 total CAUTI events to achieve 80% power atα = 0.05 Our single-center quality improvement project, constrained by a finite observation window (6-month post-intervention follow-up), accrued only 45 total events (28 pre-and 17 post-intervention), which is insufficient to detect a moderate effect with conventional statistical certainty. Thus, interpreting the non-significant *p*-value solely as “no effect” would be methodologically inappropriate; rather, it likely reflects a Type II error arising from event rarity.

Third, and most critically, all process-based surrogate endpoints exhibited robust and statistically significant improvements. Hand hygiene compliance, daily catheter-necessity assessment, and catheter utilization rate-all well-validated intermediate indicators for CAUTI prevention-improved substantially. These findings unequivocally confirm that the evidence-based localized measures were faithfully translated into clinical practice and that nursing staff adhered to the revised protocols. The observed downward trajectory in CAUTI rates, though statistically non-significant, is biologically coherent and directionally consistent with these process changes. In the field of infection control, where rare events are common, a consistent trend across multiple surrogate indicators often provides stronger evidence of intervention effectiveness than an underpowered comparison of the outcome alone. Future multi-center studies with extended follow-up periods and larger sample sizes are warranted to validate whether this clinically favorable trend achieves statistical significance.

## 6 Conclusion

In conclusion, this evidence-based localized quality improvement initiative, grounded in a rigorous evidence synthesis and Delphi-based contextual adaptation, substantially enhanced nursing process performance, as reflected by statistically significant improvements in hand hygiene, daily catheter necessity assessment, and catheter utilization rates. Although the 15.7% relative reduction in CAUTI incidence was not statistically significant-likely attributable to limited statistical power due to the low number of observed events-the direction and magnitude of the reduction are clinically encouraging and consistent with the significant improvements in process indicators. These findings underscore the value of embedding best evidence into clinical protocols and highlight the necessity of large-scale, prolonged surveillance to definitively establish the effectiveness of such interventions on rare clinical outcomes. Future multi-center studies with extended follow-up are warranted to validate the statistical significance and generalizability of these clinically favorable trends.

## Patient and public involvement

Patients or the public were not directly involved in the design, conduct, reporting, or dissemination plans of this research. However, as this study focused on quality improvement in clinical practice, the implementation of evidence-based measures inherently prioritized patient safety and outcomes. Feedback from patients and caregivers regarding catheter care experiences was indirectly considered through routine clinical evaluations and nursing audits, which informed the need for this evidence-based practice improvement initiative. The ultimate goal of this research is to enhance patient-centered care by reducing infection risks and improving healthcare quality.

## Data Availability

The original contributions presented in the study are included in the article/supplementary material, further inquiries can be directed to the corresponding authors.
